# Psychological Distress and Tobacco Use Among Hospital Workers During COVID-19

**DOI:** 10.3389/fpsyt.2021.701810

**Published:** 2021-07-28

**Authors:** Izza Mounir, Loick Menvielle, Samir Perlaza, Denis Chênevert, Jo-Hanna Planchard, Roxane Fabre, Michel Benoit, Amine Benyamina, Benjamin Rolland, Faredj Cherikh, Renaud David

**Affiliations:** ^1^Department of Addiction, University Hospital of Nice, Nice, France; ^2^Department of Human Resources Management, Edhec Business School, Nice, France; ^3^Centre de Recherche de Sophia Antipolis-Mediterranee, INRIA, Valbonne, France; ^4^Department of Human Resources Management, HEC Montreal, Montreal, QC, Canada; ^5^Department of Occupational Health, Cimiez Hospital, Nice, France; ^6^CoBTeK Lab, Department of Public Health, Côte d'Azur University, University Hospital of Nice, Nice, France; ^7^Department of Psychiatry, University Hospital of Nice, Nice, France; ^8^Department of Psychiatry and Addiction, AP-HP, Paul Brousse Hospital, Paris, France; ^9^Academic Department of Addiction Medicine in Lyon, Centre Hospitalier Le Vinatier, Hospices Civils de Lyon, Bron, France; ^10^Department of Geriatric Psychiatry, Cimiez Hospital, Nice, France

**Keywords:** COVID-19, lockdown, tobacco, physical activity, psychological distress, health behaviors, hospital workers

## Abstract

**Background and Aims:** This work aims to assess the impact of the COVID-19 pandemic on hospital workers' psychological parameters and attitudes toward substance use, before and during the French COVID-19 lockdown.

**Methods:** An online survey was proposed to the staff of the University Hospital of Nice and Sainte-Marie psychiatric hospital in France from May 18 to June 6, 2020 assessing changes in daily habits, psychological distress and changes in substance use, including smoking.

**Results:** A total of 702 respondents (80.3% female) filled out the survey. Overall, most of the workers reported increased stress, irritability, sadness, decreased motivation, and a worse quality of sleep after the beginning of the COVID-19 lockdown. Additionally, hospital workers who were more likely to use tobacco during the COVID-19 lockdown reported increased sadness (*OR* = 1.23, *p* < 0.001), loss of motivation (*OR* = 0.86, *p* < 0.05), alcohol consumption (*OR* = 3.12, *p* < 0.001), lower incomes (*OR* = 1.69, *p* < 0.05), living alone (*OR* = 1.77, *p* < 0.001) and doing less physical activity (*OR* = 0.36, *p* < 0.001).

**Conclusion:** During the first lockdown, significant psychological changes (sadness, distress, irritability) associated with changes in tobacco use and physical activity were reported. Such results should encourage hospital leaders to implement dedicated policies to better accompany hospital workers' psychological distress.

## 1. Introduction

In March 2020, the World Health Organisation declared the coronavirus disease 2019 (COVID-19) to be a pandemic, one of the most dramatic global tragedies in the twenty-first century. Since then, hospital workers have been overwhelmed, which threatens their physical health (1). In general, public health emergencies affect individuals and communities through different perspectives. Individuals are often subject to anxiety, stress, depression, loneliness, insecurity, confusion, and stigma. Communities are affected by economic losses, social distancing, mobility restrictions, lockdowns, closing businesses and schools, and insufficient resources for appropriate medical service. All these elements together lead to a sequence of emotional reactions and unhealthy behaviors such as distress, psychiatric conditions and excessive substance use (2). These was evidenced during the SARS outbreak in 2003, whose repercussions pursue even years after the epidemic (3, 4). The psychological impact of lockdown during this SARS pandemic induced higher levels of depression (5), irritability and anxiety (6). In the particular case of health care providers, alcohol abuse and dependence symptoms were reported even up to 3 years later. This population self-declared alcohol abuse as a coping strategy and was found significantly related with hyperarousal (7).

These findings ripple through populations affected by the COVID-19 pandemic and pave the way for substance abuse. In China, the psychological impact of COVID-19 is translated into severe anxiety responses present in one third of the general population (8, 9). Other studies in Spain (10), France (11) and Australia (12) reported an increment of symptoms of anxiety, depression, or irritability, which comes often together with significant augmentation of consumption of both alcohol and tobacco; the willingness to practice physical activity; and the quality of sleep.

The first national lockdown in France started on March 17 and ended on May 11, 2020. Unfortunately, the impact of these preventive measures on hospital workers is difficult to assess due to the lack of data. Nonetheless, insufficient supply of tests, limited treatment options, inadequate personal protective equipment and other medical equipment, as well as, prolonged workloads have been observed in most health centers. This may require professional mental health intervention to smooth down long term consequence on hospital workers (2). These psychological distress effects tent to aggravate when lockdowns and other social distancing measures are announced by goverments, c.f., lockdown extensions in Spain (10). In France, the COVID-19 prevention study (13) and the coronavirus and confinement longitudinal survey study (14) highlight the negative impact of the lockdown on the general population in terms of depression and other behaviors such as tobacco use, sleep disorder and weight gain. This reinforces the thesis that worsening the mental health plays a central role in the appearance of harmful lifestyle habits, such as alcohol, tobacco, sleep disorders, low quality diet and deficient physical activity (15, 16).

Within the context of COVID-19 lockdowns, groups with vulnerabilities to psychological distress effects are well identified: healthcare providers (17); people with past somatic diseases or current mental disorders, such as depression, anxiety or bipolar disorder (10); and people with substance use disorders. With the adoption of mobility restrictions and social distancing, usual places promoting physical activity and open-air activities were no longer accessible, which becomes an additional constraint to reduce psychological distress (12, 18).

Since 2014 in France, tobacco control policies have been implemented nationwide. In 2019, 30.4% of French people aged between 18 and 75 declared smoking, from which 24% with daily consumption (19). In the context of the first COVID-19 lockdown in France, our hypothesis is that hospital workers have changed their work habits; tobacco and alcohol use; and have experienced psychological distress, in terms of quality of sleep, motivation, stress, irritability and sadness.

## 2. Materials and Methods

### 2.1. Population and Data Collection

A cross-sectional survey was sent by email to the staff of the University Hospital of Nice and the Sainte-Marie psychiatric hospital, between May and June 2020. The survey was anonymous and compliant with the French general data protection regulation and local laws (20). The population's characteristics are described in Table 1.

**Table 1:** Participants' characteristics and medical history.

	**n**	**%**
**Gender**		
Female	564	80.3
Male	138	19.7
**Age**		
18–30 years	107	15.2
31–40 years	204	29.1
41–50 years	196	27.9
51–65 years	188	26.8
65 years and more	7	1.0
**Living with a partner**		
No	254	36.2
Yes	448	63.8
**Dwelling place**		
Apartment	483	68.8
House	219	31.2
**Change of dwelling during lockdown**	
No	671	95.6
Yes	31	4.4
**Decreased income**		
No	573	81.6
Yes	31	18.4
**Tobacco consumption**		
No	557	79.3
Yes	145	20.7
**Alcohol consumption**		
No	589	83.9
Yes	113	16.1
**Cardiovascular disease**		
No	655	93.3
Yes	47	6.7
**Immunosuppression**		
No	686	97.7
Yes	16	2.3
**Chronic inflammatory disease**		
No	664	94.6
Yes	38	5.4
**Pregnancy**		
No	683	97.3
Yes	19	2.7
**Obesity**		
No	649	92.5
Yes	53	7.5
**Depression**		
No	671	95.6
Yes	31	4.4
**Chronic respiratory disease**		
No	668	95.2
Yes	34	4.8

### 2.2. Measures

The data was collected via a survey that was divided into three parts. The first part aims to collect information gender, age, current living situation and medical history concerning indicators of increased risk of severe COVID-19 illness [(21); See Table 1]. The second part gathers information about substance use and physical activity. This part also collects self-evaluation data on psychological distress values (sleep, sadness, irritability, motivation, stress) before and after the lockdown in a scale from zero to five. Zero and Five represent, respectively, the lowest and highest scores in sadness, irritability, stress, and motivation. Concerning the quality of sleep, zero and five represent the worse and the best sleep quality, respectively. The third part focuses on tobacco use with a Fagerström Test for Nicotine Dependence (FTND) and the french version of the tobacco craving questionnaire (FTCQ-12) which are valid and reliable self-report instruments (22). The Fagerström test is a six-item scale standard instrument for assessing the intensity of a physical addiction to nicotine with a total score varying from 0 to 10 (23). On the other hand, the FTCQ is a twelve-item scale test designed to assess tobacco craving in four factors: emotional, expectancy, compulsion, and purposefulness (24).

### 2.3. Statistical Analysis

All the respondents (*n* = 702) were used in this survey, except for the Fagerström Test that concerned only tobacco users (*n* = 145). Percentages and frequencies, were used for categorical variables. Alternatively, the mean and the SD were used for quantitative variables. In order to test the evolution of the different scores between the period “before lockdown” and the period “after the lockdown,” paired Student's tests were used. The scores follow a Gaussian distribution, the Shapiro statistical test was used for validating this assumption. Tobacco use have been investigated as follows. A Student's *t*-test was used for the quantitative variables; and a Chi-square test was used for the qualitative variables. A multivariable logistic regression was constructed using the variables with a *p*-value smaller than 0.150 in univariate. Only variables with a p-value smaller than 0.05 were kept in the final model. Ninety-five percent confidence intervals have been shown. In the multivariable model, adjustment in *p*-value for multiple comparisons using Holm method was made with the *p*-adjust function. The analyzes were carried out using software R 3-5-1.

## 3. Results

### 3.1. Descriptives

Participants characteristics are described in the Table 1. The population in this research is representative of the one in the University Hospital of Nice given that 74.11% female workers were registered in 2019. From the whole population, 20.7% use tobacco and 16.1% use alcohol. The rate of tobacco use is lower than that observed in the French population (30.4%) aged 18–75 in 2019 but closer to daily tobacco use in France (24%) (19). In our survey, the ratio of alcohol use is also lower than the ratio of French adults in 2018, which is 87% consumption at least once per year and 49% once a week (25). Otherwise, only a few participants had to change their dwelling (4.4%) or went through a decreased income (18.4%) during the first lockdown in France.

Table 1 shows that hospital employees have a lower rate of depression (4.4%) and obesity (7.5%) than the general population in France. More specifically, according to the last survey conducted in 2017, 10% of the population has experienced a depressive episode (26) and 17% of the French population suffered obesity in 2019 (27).

### 3.2. Lockdown Impact on Tobacco Consumption

Table 2 underlines the negative impact of the lockdown on tobacco consumption for hospital employees. According to the Fagerström test, it can be concluded that the nicotine dependence had increased up to 24% after the lockdown. Hospital workers who before the lockdown were not considered as dependent or weakly dependent to nicotine became moderately or strongly dependent. More specifically, the moderately dependent population increased from 18.6% to 24.8%, whereas, the strongly dependent population increased from 3.9% to 24.8%.

**Table 2:** Impact of Fagerstöm Test related to the lockdown and correlation to FTCQ-12.

			**FTCQ-12**		
	**n**	**%**	**Mean**	**SD**	**p-value**
**Fagerström test before the lockdown**					0.094
Not addicted to nicotine	63	48.8	39.27	12.57	
Weakly dependent to nicotine	37	28.7	42.35	13.34	
Moderately dependent to nicotine	24	18.6	42.76	12.61	
Strongly dependent on nicotine	5	3.9	53.60	14.72	
**Fagerström test since the lockdown**					<0.001
Not addicted to nicotine	54	41.9	36.88	10.32	
Weakly dependent to nicotine	29	22.5	38.30	11.70	
Moderately dependent to nicotine	32	24.8	46.68	13.02	
Strongly or very heavily dependent on nicotine	14	10.9	56.85	10.81	
					<0.001
**Increased Fagerström score**	30	24.4	50.33	12.23	
**No change in Fagerström score**	88	71.5	39.08	11.78	
**Lower Fagerström score**	5	4.1	36.25	11.59	

Other studies made in France during the lockdown on the general population showed that tobacco use increased during this period. The results of this research are consistent with the french national public health agency's survey that found around a quarter of smokers (27%) had increased their tobacco consumption. Rossinot et al. (11) found that the proportion of participants who increased their consumption doubles the proportion of those who decreased it. Rolland et al. (28) reported more increases in addiction-related habits than decreases. More specifically, 35.6% of the population augmented tobacco use, from which 26.72% declared having moderately augmentation; and 8.92% declared an augmentation in a difficult-to-control manner.

To evaluate nicotine dependence with cravings, correlations between the Fagerström test and the French version of the Tobacco Craving Questionnaire (FTCQ-12) are presented in Table 2. There are no significant differences before the lockdown on nicotine dependence and tobacco craving (*p* = 0.094). After the lockdown, significantly (*p* < 0.001) higher tobacco craving scores (FTCQ-12) are observed for moderately nicotine-dependent individuals (mean = 46.68, *SD* = 13.02) compared to non-dependent individuals on nicotine (mean = 36.88, *SD* = 10.32). This implies a strong relation between a higher tobacco craving and increased scores on Fagerström test on hospital employees as a negative impact of the lockdown on tobacco use.

### 3.3. Lockdown Impact on Psychological Distress Values

The results in Table 3 show significant negative effects for all the distress values on hospital workers after the lockdown (*p* < 0.001). The respondents reported having a lower quality of sleep, a higher stress, irritability, sadness and also a loss of motivation.

**Table 3:** Impact of the lockdown on psychological distress values.

	**All population**		**Non-tobacco use**		**Tobacco use**		
	(***n*** = 702)		(***n*** = 557)		(***n*** = 145)		
	**Mean**	***SD***	**Mean**	***SD***	**Mean**	***SD***	**p-value**
**Sleep**							
Before	3.53	1.12	3.55	1.11	3.41	1.16	0.134
During	2.78	1.40	2.86	1.39	2.47	1.38	0.003
Difference	–0.75	1.40	–0.71	1.36	–0.94	1.51	0.074
**Stress**							
Before	2.23	1.27	2.20	1.27	2.36	1.27	0.175
During	2.77	1.41	2.73	1.41	2.96	1.41	0.076
Difference	0.54	1.44	0.53	1.44	0.60	1.45	0.592
**Sadness**							
Before	1.08	1.23	1.04	1.21	1.23	1.29	0.105
During	1.71	1.48	1.60	1.44	2.14	1.55	<0.001
Difference	0.63	1.28	0.56	1.25	0.92	1.38	0.003
**Motivation**							
Before	3.67	1.06	3.68	1.04	3.62	1.12	0.521
During	3.05	1.35	3.13	1.32	2.77	1.42	0.005
Difference	–0.62	1.27	–0.56	1.23	–0.85	1.38	0.014
**Irritability**							
Before	1.84	1.27	1.81	1.28	1.97	1.21	0.160
During	2.37	1.40	2.30	1.38	2.64	1.43	0.009
Difference	0.53	1.42	0.50	1.43	0.67	1.37	0.194

Table 3 compares tobacco consumers and non-consumers with the psychological distress values. Tobacco consumers face a worse quality of sleep (mean = 2.47, *SD* = 1.38, and *p* < 0.003), sadness (mean = 2.14, *SD* = 1.55, and *p* < 0.001), irritability (mean = 2.64, *SD* = 1.43, and *p* < 0.009) and went through a loss of motivation (mean = 2.77, *SD* = 1.42, and *p* < 0.005) compared to non-tobacco consumers.

### 3.4. Lockdown Impact on Physical Activity

In Table 4, the results showed a decrease in physical activity since the beginning of the lockdown. Those who were having less than 30 min of physical activity per day did not have a significant change after the lockdown. About a third of the population (33%) reported reduced exercise time since the COVID-19 lockdown. The population that did not practice any physical activity increased from 23.5% to 38% after the lockdown.

**Table 4:** Impact of the lockdown on physical activity.

	**All population**		**Non-tobacco use**		**Tobacco use**		
	***n*** = 702		***n*** = 557		***n*** = 145		
	***n***	**%**	***n***	**%**	***n***	**%**	**p-value**
**Physical activity before the lockdown**							0.001
Do not do any physical activity	165	23.5	106	19.0	59	40.7	
<30 min a day	256	34.5	208	37.3	48	33.1	
30–60 min a day	208	29.6	181	32.5	27	18.6	
>1 h a day	73	10.4	62	11.1	11	7.6	
**Physical activity since the lockdown**							0.001
Do not do any physical activity	267	38.0	192	34.5	75	51.7	
<30 min a day	260	37.0	213	38.2	47	32.4	
30–60 min a day	133	18.9	117	21.0	16	11.0	
>1 h a day	42	6.0	35	6.3	7	4.8	
**Change in physical activity**							0.020
Increased	7	11.0	64	11.5	13	9.0	
No change	393	56.0	297	53.3	96	66.2	
Decreased	232	33.0	196	35.2	36	24.8	

In Table 5, correlations between physical activities and psychological distress values are presented. Since the lockdown, hospital workers reported poorer quality of sleep (mean = −1.00, *SD* = 1.45, and *p* < 0.001), more stress (mean = 0.67, *SD* =1.46, and *p* < 0.020), loss of motivation (mean = −0.84, *SD* = 1.37, and *p* < 0.001) and irritability (mean = 0.82, *SD* = 1.40, and *p* < 0.001). This implies a significant dependence between a reduced physical activity and worsening psychological values as previous studies showed (11, 29).

**Table 5:** Impact of the lockdown on psychological distress values correlating with physical activity.

	**Physical activity since the lockdown**							
	**Increase**		**No change**		**Decrease**		
	(***n*** = 77)		(***n*** = 393)		(***n*** = 232)		
	**Mean**	***SD***	**Mean**	***SD***	**Mean**	***SD***	**p-value**
**Sleep**							
Before	3.35	1.01	3.55	1.04	3.56	1.00	0.325
During	3.00	1.36	2.84	1.40	2.56	1.37	0.006
Difference	–0.25	1.64	–0.71	1.27	–1.00	1.45	<0.001
**Stress**							
Before	2.34	1.07	2.19	1.25	2.26	1.34	0.584
During	2.48	1.46	2.74	1.42	2.94	1.37	0.036
Difference	0.14	1.63	0.54	1.38	0.67	1.46	<0.020
**Sadness**							
Before	1.03	1.19	1.08	1.20	1.10	1.30	0.889
During	1.58	1.50	1.65	1.44	1.86	1.53	0.179
Difference	0.56	1.38	0.58	1.25	0.75	1.31	0.216
**Motivation**							
Before	3.29	1.23	3.66	1.07	3.81	0.94	0.001
During	3.08	1.29	3.16	1.36	2.87	1.35	0.037
Difference	–0.21	1.25	–0.50	1.17	–0.94	1.37	<0.001
**Irritability**							
Before	1.90	1.27	1.86	1.26	1.79	1.29	0.732
During	2.12	1.39	2.28	1.39	2.61	1.38	0.005
Difference	0.22	1.64	0.42	1.35	0.82	1.40	<0.001

Table 4 shows a significant negative association between tobacco consumers and physical activity. Tobacco users exercise less than non-users. For instance, before the lockdown, the population of smokers that do not practice any physical activity was 40.7%, whereas the nonsmoking counterpart was 19%. After the lockdown, these populations increased to 51% and 34.5%, respectively.

Given the psychological distress responses to COVID-19 lockdown shown before and the demonstrated benefits of physical activity over behavioral distress (30), further strategies to promote physical activity for hospital employees are required.

### 3.5. Multivariable Analysis

Table 6 shows the results of the adjusted OR of some independent variables with tobacco consumers. The profile that was identified to be the most at risk of increasing tobacco use corresponds to the population that satisfies the following criteria: (*a*) living alone [aOR 1.77, 95% CI (1.18–2.65) and *p* < 0.005]; (*b*) experiencing increased sadness [aOR 1.23, 95% CI (1.08–1.41), and *p* < 0.002]; (*c*) experiencing loss of motivation [aOR 0.86, 95% CI (0.74–1.00), and *p* < 0.043]; (*d*) receiving a lower income [aOR 1.69, 95% CI (1.04–2.70), and *p* < 0.031]; (*e*) consuming alcohol [aOR 3.12. 95% CI (1.92–5.05), and *p* < 0.001]; and (*f*) reducing physical activity [aOR 0.25, 95% CI (0.14–0.42), and *p* < 0.001].

**Table 6:** Multivariable analysis on increasing the tobacco use related to the lockdown.

	**OR adj**	**[CI 95%]**	**p-value**	**p-value adj**
Sadness during	1.23	[1.08; 1.41]	0.002	0.010
Motivation during	0.86	[0.74; 1.00]	0.043	0.063
Living alone	1.77	[1.18; 2.65]	0.005	0.016
Physical activity before the lockdown				
<30 min a day	0.36	[0.22; 0.58]	<0.001	<0.001
30–60 min a day	0.22	[[0.10; 0.45]	<0.001	<0.001
>1 h a day	0.25	[0.14; 0.42]	<0.001	<0.001
Having a lower income	1.69	[1.04; 2.70]	<0.031	0.063
Consuming alcohol	3.12	[1.92; 5.05]	<0.001	<0.001

*The *p*-value is shown in two columns. The second column shows the correction for multiple comparisons with Holm's method*.

## 4. Discussion

The present research examined the association between the first COVID-19 lockdown and the change in health behavior of hospital workers. The overarching conclusion is that the lockdown and the associated restrictive measures had a negative effect on this population. All aspects of psychological distress (sleep, sadness, motivation, irritability and stress) were significantly associated with changes in health behaviors (tobacco use and physical activity).

One of the main conclusions of this work is that living alone is a major risk factor for mental health issues and increased tobacco use. Similar results were found in other studies in the general french population. Rolland et al. (28) identified that being a female; not having a partner; being professionally active; and having a relatively low level of education are conditions associated with higher risk for increasing tobacco use. Rossinot et al. (11) described living alone as a strong risk factor for diet, sleep, physical activity, and tobacco consumption. In other countries, living alone has statistically strong significance with increased smoking during the lockdown. In Australia, Stanton et al. (12) showed that those who were not in a relationship had significantly higher depression, anxiety and stress scores compared to other categories of relationship status.

An interesting observation from the data obtained during this research is that participants that reported tobacco use, also reported lower scores in sleep quality and motivation; and higher scores in sadness and irritability. Similar observations have been reported in studies concerning the association between health-related behaviors and psychological distress factors. During the lockdown in France, Rossinot et al. (11) reported that deterioration on mental health (symptoms of anxiety, depression, or irritability) translates into worsening most behavioral indicators (tobacco and alcohol consumption, physical activity and sleep). These results are in concordance with the findings of the coronavirus and confinement longitudinal study (14) and the COVID-19 prevention studies in France (13). This observation has been made in the general population of other countries (12, 31–33).

It is important to highlight that the number of cigarettes smoked per day heavily increased withing the sampled population. This is justified by higher Fagerström scores in our survey. In the general population in Belgium (34), the odds of increasing tobacco use has doubled. In Poland, Sidor and Rzymski (35) conducted an online survey among 1097 adults during the lockdown showing that 45.2% of smokers had augmented their tobacco consumption. In China, Sun et al. (36) led an online survey among 6.416 adults and found that 20% of regular smokers had increased their tobacco use and 25.3% of ex-smokers had relapsed.

Another observation is that hospital staff decreased their physical activity during the lockdown. This decrease was more significant for tobacco users. One explanation is that during health crisis, hospital workers endure longer working schedules and increased tensions due to important responsibilities (9). This observation concurs with previous studies (12, 29) on general populations.

The above discussion highlights the behavioral response of hospital workers in times of stress and may, therefore, be valuable for providing the appropriate care and preventive actions in case of similar future events. This might include screenings for mental health problems, substance abuse, psychoeducation, and psychosocial support. Other alternatives include adaptation strategies and resilience capacities based on physical activities, sleep management, substance use control, increase motivation, integrate behavior change techniques, coaching, mindfullness groups, and counseling services on mental health.

### 4.1. Limitation and Research Perspective

This survey is based on participants' self-assessment on different parameters. The data analysis takes into account the subjectivity of these evaluations. More specifically, this survey aimed to capture participants' perceptions, feelings, and views on the impact of the COVID-19 pandemic and the associated lockdown. From this perspective, answers are assumed to be correct. Methods for analyzing data in which correctness is not the main assumption are presented in (37).

The population was composed by a majority of women. Nevertheless, this is in agreement with the women population working in the University Hospital of Nice (74% in 2019) and the fact that women respond more often than men to surveys is a well-known phenomenon. Therefore, this bias is not specific to this survey. The rate of tobacco smokers in our study (20.7%) was close to that observed in the general french population (24% in 2019) (19).

A limitation of this work is that the data encompasses only the first phase of the lockdown, and thus, long-term behaviors might not be accounted on this study.

Decision-making actions aiming to help hospital workers' health might benefit from tools capable of providing real-time monitoring. For instance, artificial intelligence can be used to gather data and guide adapted interventions aiming to anticipate negative effects. We believe that the results of our survey could help health systems and policymakers to better manage new waves of the pandemic, and the post-COVID-19 period.

### 4.2. Conclusion

In conclusion, our data suggests that negative changes in health behaviors are associated with increased psychological distress in hospital workers during the COVID-19 lockdown. Tobacco consumers are having increased psychological distress than their non-consuming counterparts. Health promotion strategies aimed at embracing or preserving positive health behaviors should go toward reducing critical and chronic increases in psychological distress during these unparalleled times. Ongoing assessment of the impact of lockdown and social distancing on health behaviors is needed to shape targeted health promotion strategies.

## 5. Comparison With Prior Work

To our knowledge, no previous study has assessed the impact of a national COVID-19 containment measure on psychological distress values, physical activity and substance use on hospital workers. This work provides the first data analysis in this population.

## Data Availability Statement

The raw data supporting the conclusions of this article will be made available by the authors, without undue reservation.

## Author Contributions

IM, FC, RD, and SP participated in the design of the article, were involved in data synthesis, data interpretation, and drafted the manuscript. RF performed the statistical analysis. All authors read and approved the final manuscript.

## Conflict of Interest

The authors declare that the research was conducted in the absence of any commercial or financial relationships that could be construed as a potential conflict of interest.

## Publisher's Note

All claims expressed in this article are solely those of the authors and do not necessarily represent those of their affiliated organizations, or those of the publisher, the editors and the reviewers. Any product that may be evaluated in this article, or claim that may be made by its manufacturer, is not guaranteed or endorsed by the publisher.
